# Occupational Exposure to Blood and Body Fluids among Health Care Workers in Teaching Hospitals in Tehran, Iran

**Published:** 2012-07-30

**Authors:** Sh Shokuhi, L Gachkar, I Alavi-Darazam, P Yuhanaee, M Sajadi

**Affiliations:** 1 Department of Infectious Disease, Loghman Hospital, Shahid Beheshti University of Medical Sciences, Tehran, Iran; 2 Infectious Diseases and Tropical Medicine Research Center (IDTMRC), Shahid Beheshti University of Medical Sciences, Tehran, Iran; 3 National research Institute of Tuberculosis and Lung Diseases, Shahid Beheshti University of Medical Sciences, Tehran, Iran; 4 Institute of Human Virology, University of Maryland, School of Medicine, Baltimore, USA

**Keywords:** Needlestick injuries, Health care workers, Blood borne pathogens

## Abstract

**Background:**

Health care workers (HCWs) are vulnerable populations for infection with blood borne pathogens. This study was conducted to determine occupational exposure to blood and body fluids among HCWs in teaching hospitals in Tehran, Iran.

**Methods:**

A self-structures questionnaire was used to study 650 HCWs during 2006 -2007 in some teaching hospitals in Tehran, Iran.

**Results:**

occupational exposure to blood and body fluids to blood and body fluids of patients was noticed in 53.4%. Recapping was the most common cause of niddle stick injuries (26.5%) and 19.9% of HCWs with a history of needlestick or mucosal exposure had sought medical advice from a specialist, 79.4% of these visited a doctor in the first 24 hours after exposure. Twenty percent of people with a history of needlestick or mucosal exposure to human immune deficiency virus positive (HIV^+^) patients received post-exposure prophylaxis and 46.7% tested themselves for seroconversion. 25.8% of HCWs with a history of needlestick or mucosal exposure with HBsAg^+^ patients received hepatitis B immunoglobuline (HBIG), all of these had received it in the first 72 hours after exposure. History of vaccination, and reassurance about the effective serum antibody titer was the most frequent reason mentioned in case the individuals did not receive HBIG (56.5%).

**Conclusion:**

There is a need for further research to investigate why many HCWs do not take prophylactic and essential actions after needle stick or mucosal exposure to body fluids of infected patients.

## Introduction

Accidental injuries caused by sharp instruments and mucosal exposure to blood and body fluids of the patients present a high risk for health care workers (HCWs). These incidents potentially predispose HCWs to infection with blood borne pathogens (BBPs), the most important of which are hepatitis c virus (HCV), hepatitis B virus (HBV) and human immune deficiency virus (HIV).[1]

The American Centre for Disease Control (CDC) has estimated an annual rate of 385,000 needlestick and sharp injuries showing an increasing trend.[2] In November 2002, the World Health Organization (WHO) reported that 2.5% of HIV contaminations as well as 40% of HBV and HCV positive cases among HCWs resulted due to occupational exposures.[3]

Lack of documentation and reporting either the cutaneous injuries caused by sharp instruments or mucosal exposure to patients' body fluids are considered as a major pitfall to protect the HCWs. Such an attitude mainly resulted of lack of knowledge regarding the importance of the issue, the wrong belief of the individual that he is knowledgeable enough to handle the case, the presupposition that no follow up and support will be provided by the management, and fear of losing situation.[4][5] This eventually results in deprivation from receiving care and the necessary treatments, the efficacy of which have been proven beneficial. For instance, the post-exposure prophylaxis (PEP) for HIV was found to be effective in approximately 80% of cases.[6]

Considering the increasing rate of blood borne infections and the economical and psychological burden of occupational exposure to the blood and body fluids of the patients, highlight the immediate need for provisional training of the HCWs. Such training includes awareness of the mechanisms leading to the infection, preventive measures, and the proper management of post exposure problem. Furthermore, safe equipment and protective instruments should be provided to all HCWs, and their utilization should even be made mandatory.[4][7]

This study was conducted to determine the prevalence rate of cutaneous injuries caused by sharp objects as well as mucosal exposure to blood and body fluids of the patients and to identify factors including clinical activities, the rate of utilization of protective tools, and the reaction of the HCWs in the event of any incident, among the HCWs in Tehran, Iran.

## Materials and Methods

A questionnaire consisting of items addressing the occupation of the individuals, history of occupational injuries caused by sharp objects and splashes of patients' blood and body fluids on the mucosal membranes, activities leading to the incident, utilization of protective tools, and the kind of practice performed by the individual after the incident were used for this descriptive study. “Cutaneous injuries”, wherever mentioned in this study, are considered as any needlestick caused by a sharp object contaminated with blood or other body fluids regardless of presence or absence of frank bleeding. “Fluids” mainly mean the body fluids potentially capable to transfer the BBPs. Any other fluid is also taken into consideration in case it is visibly contaminated with blood.

The sample population of the study consists of residents, interns, nurses, and nurse aids of different wards of teaching hospitals affiliated to Shahid Beheshti University of Medical sciences in Tehran, Capital of Iran. A total of 650 HCWs were selected randomly by a combination of stratified (various hospitals, departments and wards) and simple random sampling (within aforementioned groups) methods. The findings of the study were analyzed by descriptive statistics by SPSS software (Version 15, Chicago, IL, USA). Chi ^2^ (or Fischer Exact) test was used for the analysis of the variables. P<0.05 was considered significant.

## Results

**Fig. 1:**
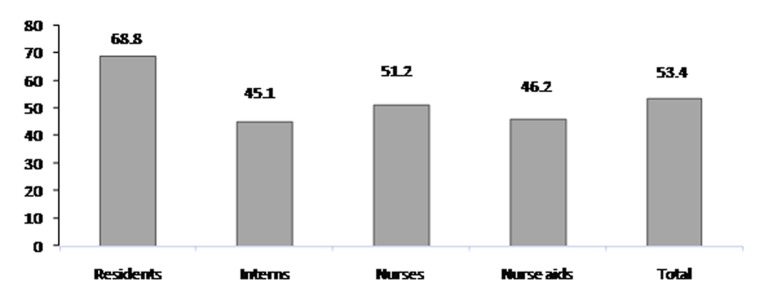
Prevalence of needlestick injuries and/ or mucosal exposure to patients' body fluids among health care workers.

Among the 650 participants, 170 (26.2%) were residents, 195 (30%) medical interns, 207 (31.8%) nurses, and 78 (12%) were nurse aids while 53.4% of them reported a needlestick injury (NSI) and/or mucosal exposure to patients' body fluids (Figure 1). The frequency of exposures was different among four groups (p<0.001). In addition, 60.3% of the participants reported more than one exposure.

Recapping the needle was reported by 76 (26.5%), suturing by 71 (24.7%) and intravenous (IV) catheter insertion by 70 (24.4%) individuals. Involvement in surgical procedures was seen in 47 (16.4%), and blood sampling in 42 (14.6%) cases as the most prevalent causes. The most prevalent causes differed among different subgroups while among residents included involvement in surgical procedures (43.6%), suturing (37.6%), and recapping the needles (15.8%); but medical interns mentioned suturing (50.8%), arterial blood gas sampling (26.2%), and recapping (15.4%). Meanwhile IV catheter insertion (42.2%), recapping the needle (40%), and blood sampling ( 24.4%) were recognized to be the most prevalent causes of the injuries among nurses which was similar to nurse-aids (IV catheter insertion, recapping the needles, and blood sampling as the most prevalent causes with prevalence rate of 58.1%, 45.2%, and 35.5%, respectively).

The restlessness and movements during the procedure was a factor contributing to at least one of the dangers (24.4%). The history of body fluids splash on their own mucosal membranes was shown in 181 (27.8%) of the HCWs (40% of the residents, 24.1% of the medical interns, 24.6% of the nurses, and 19.2% of the nurse aids). The prevalence rate of body fluids splashes on the mucosal membranes among the different subgroups was not different.

Surgical procedures, suturing, IV catheter insertion, and removing the IV catheters were recognized as the most prevalent causes of body fluids splashes on the mucosal membranes.

Protective equipments such as masks, glasses, and shields have been used in 35.9% of these individuals (70.6%, 27.6%, 5.9%, and 6.7% of the residents, medical interns, nurses, and nurse-aids, respectively) (p<0.001); 91.2% of the individuals with a history of body fluids splash had mucosal exposure as a result of ignoring the utilization of the protective equipments or improper utilization.

Among HCWs with a history of either cutaneous injury or mucosal exposure to body fluids of the patients, 19.9% (17.1% of the residents, 21.6% of medical interns, 20.7% of the nurses as well as 22.2% of the nurse- aids, p>0.05) were referred to a specialist (internal medicine or infectious diseases) while 79.4% of these referrals occurred within 24 hours of exposure. Forty percent of the HCWs with a history of needle stick or mucosal exposure to body fluids of the patients noted that they had tried to assess the situation based on their own knowledge and expertise. This included 54.7% of the residents, 30.7% of the medical interns, 43.4% of the nurses, and 11.1% of the nurse-aids (p<0.001); 23.4% of them (18.7%, 25.9%, 23.9%, and 75% of the residents, medical interns, nurses, and nurse- aids, respectively) never reviewed the profile of the source patients (p>0.05). In order to find out the appropriate approach to the problem, 14.4% of the exposed population referred to the textbooks. This included 19.4% of the residents as well as 15.9% of the medical interns and 2.8% of the nurses. None of the nurse-aids referred to the textbooks.

In 54.5% of the HCWs with a positive history of either needle stick or mucosal exposure (60.7% of the residents, 47.7% of the medical interns, 59.5% of the nurses, and 36.1% of the nurse- aids), the profile of the patients who had been the source of contamination regarding blood borne viral infections were reviewed (p<0.003); 5.8% (3 cases) of them had noted that their patient was infected with HIV, while 38.4% (20) had patients with HBV, 23.1% (12) had patients with HCV, and 11.6% (6) were infected with HIV and HCV at the same time. In addition, 9.6% (5) had mentioned their patients had both HBV and HCV; and 11.5% (6) had noted that the patients had a triple infection.

Only 20% of the HCWs whose patients were HIV^+^ had received PEP (two residents and 1 nurse; and none of the 5 exposed medical interns had received PEP (p>0.05). Considering the onset of the treatment, the number of medications, and their types, it seemed that PEP was conducted properly in the residents, while the only exposed nurse did not receive the proper prophylaxis. Among exposures to HIV^+^ patients, 46.7% of the HCWs reviewed their own seroconversion; only one of them was followed properly. There was no significant difference among the subgroups in their seroconversion assessment.

Among exposures to HBsAg^+^ patients, 25.8% of the exposed HCWs (22.2% of the medical interns, 71.4% of the nurses, and 20% of the nurse-aids) received hepatitis B immunoglobuline (HBIG) during 72 hours after exposure; none of the residents received HBIG (p<0.001). History of vaccination, and reassurance about the effective serum antibody titer was the most frequent reason mentioned in case the individuals did not receive HBIG (56.5%).

## Discussion

Unlike the previous studies in which nurses were recognized as the most vulnerable group as a result of their intimate exposure to the patients,5 this study depicted residents as the subgroup with the highest rate of exposure. In United States, nurses ranked first (49.7%), and then the group of medical doctors (12.6%). Nurses were also recognized as the most vulnerable group in Canada (70%).[8][9] The different rates reported in present study might have been resulted from the different occupational categories studied. The results of the studies which have introduced residents of surgery as the most vulnerable group, even ranking higher than surgery attending staff, may shed light on the differences observed in the present study.[10] Deeming the fact that 45.9% of the residents participating in our study were from different disciplines of surgery, one can justify the higher rate of the exposure in this group. The prevalence rate of NSIs has been 33.3% among our medical interns. In the French study,[11] 25% of the medical students have reported to have received at least one NSI while the American study has reported the same figure to be 30%.[12] These results are compatible with the findings of the present study. The prevalence among the nursing workers (nurses and nurse-aids) has been 42.5% which complies with the results of the study conducted in 2001 in Yasuj District of Iran where the prevalence rate was reported as 46.2%.[13]

In this study, needle recapping, suturing, IV catheter insertion, surgical procedures, and blood sampling have been respectively recognized as the most prevalent causes of the injury. In the study conducted in Yasuj District hospitals in 2001, the most prevalent etiologies were reported as IV catheter insertion, injections, and blood sampling.[13] Meanwhile, the results of the 2004 study in Kordestan University of Medical Sciences, Iran introduced injection/blood sampling, surgical procedures and disposal of either the needle or angio-catheter as the most prevalent causes.[14] Even though recapping has not been mentioned in these studies, it can be concluded that the existing differences with our study results from the different samples studied in the studies.

Kamali and Motamedi (2001) have found recapping as the main reason for NSIs, a result which is compatible to the results of the present study.[15] The Australian study has considered recapping as an integral part of other procedures and has introduced hollow bored needles as the main source of injury which is compatible with our results.[16] The same study highlights that majority of the NSIs had been caused through intramuscular or subcutaneous injections, intravenous injections, and arterial or venous blood sampling while in our study, the main causes included IV catheter insertion, blood sampling, and injection of medications. The different ranges of the activities conducted by the sample populations enrolled in the two studies clarify the existing difference between the results. NSIs caused by solid sharp objects are mainly a result of injuries occurring in the process of suturing and applying the surgical scalpels which is compatible with ours. The Massachusetts report (2004) notes injection, suturing, blood sampling, and IV catheter insertion as the main causes of injuries.[17] The existing differences between the two studies might have been resulted from the different occupational subgroups enrolled and the fact that recapping the needles is not a common practice in the United States. Hence, one can not consider a definite activity as the activity most probably accompanied by the injury and the cause of the injury is also correlated to occupation and some other factors. Overall, it seems that recapping lies among the most prevalent causes.

The order of the activities resulting in injury among the population participating in this study has been different in the various occupational subgroups. Among the medical interns, the most prevalent causes included suturing, arterial blood gas sampling, recapping the needles and ascites fluid puncture. The study was conducted in Tehran University of Medical Sciences, where data were collected from the medical interns, indicated that the most prevalent causes for the injury included injection or phlebotomy (29%), suturing (27.9%), arterial puncture (15.3%), and pleural or ascitis fluid puncture (7.9%).[18] This study has also included recapping as an integral part of the process of other activities performed by the individuals. Taking this point into consideration, and putting injection and phlebotomy aside, as they are not a part of the routine of the medical interns in our sampling population, the order of the prevalence of the different causes of injury in this study seem to be consistent with our results.

24.4% of the cutaneous injuries of our HCWs had somehow resulted of restlessness or movement of the patient. The Massachusetts report (2010) has noted that 5% of the injuries were the result of the movement of the patient.[17] Thus, such causes should be considered as factors playing a major role in incidence of cutaneous lesions by sharp objects, and preventive measures must be highly considered. More frequent use of protective equipment by residents as compared to other groups might have its roots in different occupational tasks undertaken. As almost half of the residents were enrolled in different categories of surgery residency programs, higher utilization rate can be justified.

On time refers (time when there was still a chance to receive HIV PEP) had occurred in 79.4% of the referrals to the specialists. Residents had the lowest rate for referral and the highest rate for self-assessment of the problem while nurse-aids had the highest rate of visiting a specialist and at the same time lowest rate of self-assessment. This might be the result of the residents' confidence about their own knowledge and efficacy in managing the crisis.

The higher rate of evaluation of the source patient by residents versus the lower rate of evaluation by nurse-aids can be attributed to the level of the knowledge of the individuals regarding BBPs and the risk of infection through occupational contacts. 47.7% of the medical interns had evaluated either all or some of their source patients. The Toronto study in 2003 has reported that 41% of the medical students evaluated their patients' profile. This result is almost compatible with ours.[19] Only 20% of the HCWs in need of PEP had received it, and 66.7% of them had received a standard treatment. In a study performed in Poland (1995-2001), 64.3% of the HCWs in need of PEP had received it.[20] Among the HCWs with a HBs Ag^+^ patient, nurses ranked the highest in receiving HBIG while residents had the lowest rate. The most prominent underlying cause for skipping HBIG was mentioned to be history of proper vaccination and having reassurance about efficient serum antibody titer (56.5%)

A significant percentage of the enrolled population who were all among HCWs have mentioned the history of needlestick and/or mucosal exposure to the body fluids of the patients and majority had not taken the necessary measures after their contact. High rate of exposures and interestingly not to evaluate and follow, a proper guideline revealed poor attitude and practice and probably knowledge about exposure classifications and attention to the associated risks. The underlying reasons are to be evaluated in further studies.
